# How to lose a hand: Sensory updating drives disembodiment

**DOI:** 10.3758/s13423-020-01854-0

**Published:** 2020-12-09

**Authors:** Roland Pfister, Annika L. Klaffehn, Andreas Kalckert, Wilfried Kunde, David Dignath

**Affiliations:** 1 Department of Psychology III, University of Würzburg, Röntgenring 11, D-97070 Würzburg, Germany; 2 Department of Psychology, University of Skövde, Skövde, Sweden; 3 Department of Psychology, University of Tübingen, Tübingen, Germany

**Keywords:** Body representation, Embodiment, Disembodiment, Moving rubber-hand illusion

## Abstract

Body representations are readily expanded based on sensorimotor experience. A dynamic view of body representations, however, holds that these representations cannot only be expanded but that they can also be narrowed down by disembodying elements of the body representation that are no longer warranted. Here we induced illusory ownership in terms of a moving rubber hand illusion and studied the maintenance of this illusion across different conditions. We observed ownership experience to decrease gradually unless participants continued to receive confirmatory multisensory input. Moreover, a single instance of multisensory mismatch – a hammer striking the rubber hand but not the real hand – triggered substantial and immediate disembodiment. Together, these findings support and extend previous theoretical efforts to model body representations through basic mechanisms of multisensory integration. They further support an updating model suggesting that embodied entities fade from the body representation if they are not refreshed continuously.

**Supplementary Information:**

The online version contains supplementary material available at 10.3758/s13423-020-01854-0.

## Introduction

Changing the physical appearance of the body is not easy, as most people will agree when thinking about their New Year´s resolutions. However, changing the mental representation of our body is surprisingly flexible and dynamic (Botvinick & Cohen, 1998). While there is good evidence that we quickly learn to acquire alien body parts (e.g., Ma & Hommel, 2015), it is less clear what happens to these representations after acquisition. The present experiment targets the maintenance of body representations in precisely these situations.

Support for the view that our body representation is not fixed, but malleable, comes from multisensory illusions, such as the rubber-hand illusion. Here participants view a rubber hand being stroked while their own hand is hidden from view. If the participants’ hand is stroked synchronously with the rubber hand, participants report vivid feelings of body ownership for the rubber hand (see also Hohwy & Paton, 2010; Moseley et al., 2008; Rohde, Di Luca, & Ernst, 2011). Similar phenomena of embodying an alien body part can be observed when multisensory synchronicity is introduced by simultaneous movements of a real and a rubber hand (Kalckert & Ehrsson, 2012; see also IJsselsteijn, Kort, & Haans, 2006; Perez-Marcos, Sanchez-Vives, & Slater, 2012; Slater, Perez-Marcos, Ehrsson, & Sanchez-Vives, 2008; Tsakiris, Prabhu, & Haggard, 2006; Yuan & Steed, 2010).

Given this remarkable flexibility, how does the cognitive system maintain a coherent body representation over time? Here we propose and assess two models to describe the fate of newly embodied parts of the body representation. On the one hand, the cognitive system may tend to retain previously incorporated entities in the body representation, as is, for example, suggested by feelings of phantom limbs after amputation (Flor et al., 1995; Ramachandran, Rogers-Ramachandran, & Cobb, 1995). A *persistence model* builds on these findings and assumes that newly embodied information remains integrated in the body representation unless incoming information actively contradicts the perception of an entity as belonging to the body (Preston & Newport, 2011), or by removing the entity altogether (Newport & Gilpin, 2011; Perepelkina et al., 2018). An *updating model*, by contrast, builds on theories that describe the body representation as a set of multisensory bindings (Blanke, 2012; Hoffmann et al., 2010; Maravita, Spence, & Driver, 2003; Tsakiris, 2017). Because such bindings decay over time, the embodied entity is assumed to fade from the body representation unless its integrated status is refreshed continually.

We compared both models using an active version of the rubber-hand illusion by asking our participants to move their real, hidden hand, which was connected to a rubber hand on the table (Kalckert & Ehrsson, 2012). We assessed the temporal dynamics of body ownership by subjective reports and compared disembodiment across three different experimental conditions. In all conditions, participants were first given the opportunity to embody the rubber hand by performing tapping movements with their index finger while observing the rubber hand tap synchronously. This procedure generates a sensation of ownership of the rubber hand because the visual appearance of the moving hand conforms to proprioceptive changes due to the participants’ finger movements (supported by an anatomically plausible orientation of the rubber hand; e.g., Fusco et al., 2020; Monti et al., 2020; Tieri et al., 2015a; Tieri et al., 2015b). Afterwards, we applied one of three manipulations. In the active condition, participants continued the tapping movements as in the initial embodiment phase. In the no-movement condition, participants stopped moving and the rubber hand remained still. Finally, in the disruption condition, the experimenter struck the rubber hand with a hammer while the participants’ real hand remained untouched.

The persistence model and the updating model both predict instant disembodiment of the rubber hand in the disruption condition, because hitting the hand provides information that actively contradicts the previous illusory experience (as suggested by the persistence model) and allows for an actualization of current multisensory bindings at the same time (as predicted by the updating model). Crucially, both models yield different predictions for the no-movement condition. Here, the persistence model suggests that the no-movement condition maintains a similarly high level of embodiment to the active condition, because neither of the two conditions provides contradictory information. In contrast, the updating model predicts gradual fading of the illusion in the no-movement condition relative to the active condition, because multisensory binding should decay over time in the former condition, but receive constant updating in the latter condition.

## Methods

### Participants

We collected data of 42 voluntary participants at the University of Würzburg. Most participants came from the local community and were not enrolled in psychology or a related degree program. The sample size was based on a pilot experiment and ensured a power of .87–.90 to detect the expected effect with null-hypothesis significance testing, even with an expected dropout rate of about 20% non-responders (see the Supplementary Material for a description and analysis of the pilot experiment; corresponding results are shown in Fig. S1).

Following our preregistration (https://osf.io/35tsv), we excluded non-responders who reported an embodiment score of 3/10 or less after the embodiment phase at least twice during the experiment (ten participants; 23.81%). This is in line with other reports that showed that about 20–30% of participants do not report a clear illusion experience (Kalckert, Bico, & Fong, 2019). The remaining sample comprised 25 female and seven male participants, all but five right-handed with a mean age of 29 years (range 19–62 years, SD = 10.09). They gave written informed consent prior to participation, and the experimenter was naïve with regard to the hypotheses underlying this study.

### Setup

**Fig. 1:**
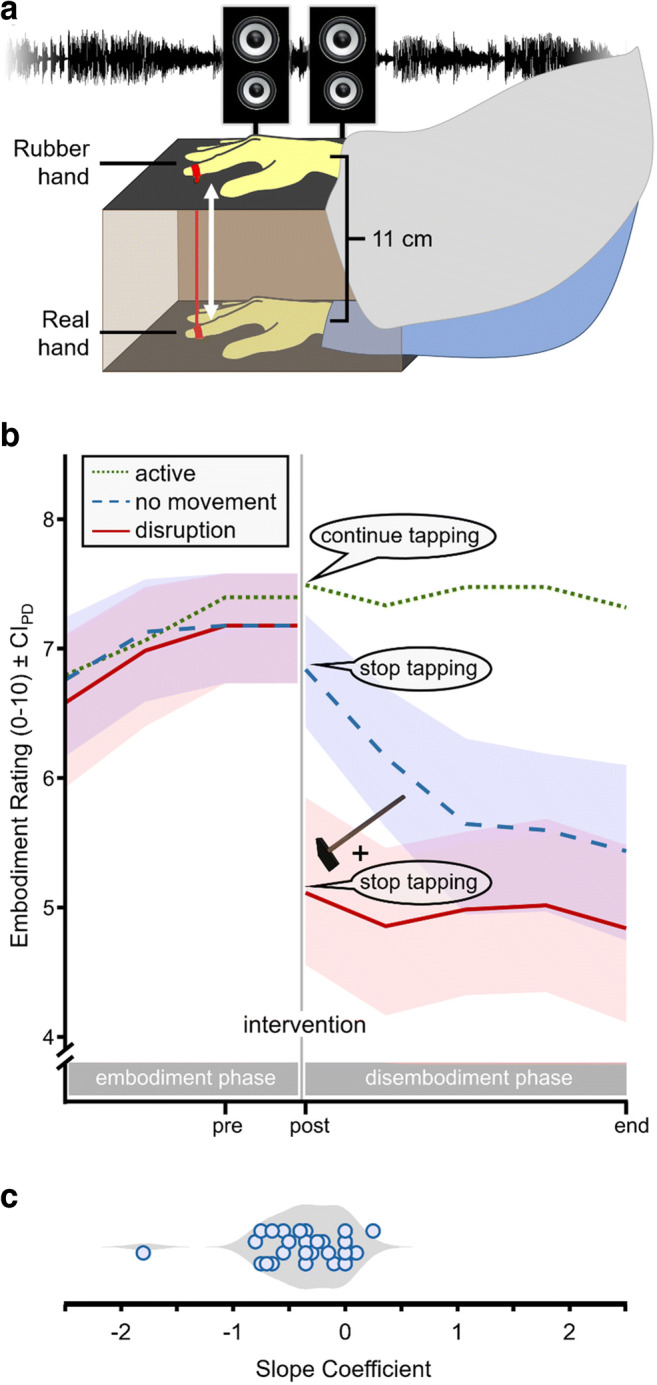
Setup and main results. (**A**) Participants inserted their right hand into an opaque box. They observed a rubber hand on top of the box and were instructed to tap to a beat. Connected rings were attached to both index fingers so that the rubber hand mimicked the participant’s tapping movements. (**B**) Mean responses to the embodiment question (rating scale ranging from 0 to 10) during the embodiment phase (three ratings) and the disembodiment phase (five ratings) in all three conditions. **Active condition**: Participants continued tapping during the disembodiment phase. **No-movement condition:** Participants stopped tapping after the intervention. **Disruption condition:** The index finger of the rubber hand was struck by a hammer once at the intervention and participants were asked to stop tapping thereafter. Ratings were separated by 30-s intervals and the first rating was preceded by a 30-s interval of synchronous tapping. Shaded areas indicate 95% confidence intervals of paired differences (CI_PD_) relative to the active condition (Pfister & Janczyk, 2013). (**C**) Individual slope coefficients when modelling the within-participant dynamics of the no-movement condition as a linear decay function. The gray area shows the fitted distribution density

The general setup was that of a moving rubber-hand paradigm (Kalckert & Ehrsson, 2012). Participants sat at a table with their right hand placed inside a box that was open towards the participant. Their index finger was inserted into a ring that was connected to a rubber hand on top of the box (see Fig. 1A; distance between real and rubber hand: 11 cm). The real and the rubber hand both wore yellow rubber gloves. The space between the rubber hand and the participant’s shoulder was covered with a blanket to hide the gap between the rubber hand and the participant’s body.

### Procedure

Participants were acquainted with the experimental setup verbally and in written form. This included an explanation of the one-item embodiment question that we used during the whole procedure. That is, participants were asked to rate on a Likert scale from 0 to 10 how strongly they felt as if the hand in their view was part of their body. Semantic anchors were provided as 0: *I feel no relation between myself and the hand*; 3: *I could imagine that the hand belongs to me*; 7: *I have the feeling that the hand is part of my body*; 10: *I have the feeling that the hand is my own hand*. Both the question as well as the anchors were presented in German language (see the Supplementary Material for the original wording).

Individual trials consisted of a 2-min embodiment phase and a 2-min phase to investigate disembodiment (“disembodiment phase”), separated by an intervention. The rubber hand apparatus was covered at the beginning of each trial. The experimenter asked the participant to close his or her eyes and inserted the hand of the participant in the apparatus, uncovered the rubber hand, and asked the participant to open their eyes again. The experimenter then started a playback of instrumental music with a distinctive beat (taken from the Epic Rap Battles of History) and prompted the participant to start tapping along with their right index finger while keeping the rest of their hand still. They were asked to watch the hand on top of the box during the whole trial. Every 30 s, the experimenter asked the participant to give their rating verbally from 0 to 10 and noted the response. After 2 min, the experimenter performed an intervention by asking participants to (a) continue tapping as before (active condition); or (b) stop tapping and keep their hand still for the rest of the trial (no-movement condition); or (c) she hit the rubber hand with a small hammer and asked the participant to keep their hand still for the rest of the trial (disruption condition). For the hammer intervention, the experimenter performed one hit on the index finger of the rubber hand and placed the hammer to the side.^1^ After the intervention, the experimenter continued to ask the embodiment question every 30 s for another 2 min (disembodiment phase). This timescale was considered appropriate for assessing decay of multisensory bindings as multisensory after-effects typically emerge on a timescale of several seconds up to minutes (e.g., Radeau & Bertelson, 1974; Tsakiris & Haggard, 2005). In between trials, participants had to take a break of at least 30 s, during which they were asked to take their hand out of the box, look at it, and move it to completely break the illusion. The rubber hand was covered in the meantime. Participants performed two identical triplets of all three conditions to improve the reliability of the measurements as compared to the pilot study, resulting in six trials overall. The order of conditions within the triplets was counterbalanced across participants, and for each participant we averaged across the two ratings of each condition for all reported analyses.

## Results

### Statistical analyses

Raw data are available online (https://osf.io/ew6k9/) and all analyses were performed as preregistered. For inferential statistics, we relied on classical frequentist approaches whenever we had predicted a between-condition difference whereas we employed Bayesian approaches whenever we had predicted equality of conditions. We further report both types of test if one of our two hypotheses predicted a difference whereas the other predicted equality of conditions. This approach ensured that our study came with the intended power, as our power calculations relied on a frequentist framework, while at the same time allowing us to quantify evidence for the null hypothesis of no difference between conditions, for which Bayesian approaches provide clear decision thresholds.

Frequentist analyses were performed with SPSS 24 whereas Bayesian approaches relied on the JASP 0.10.2, using a scale parameter of 0.5 for fixed-effects terms in ANOVA models and $\sqrt{2}$ as a conservative prior for our expected effect sizes for Bayesian *t*-tests.

### Omnibus tests

Figure 1B shows the participants’ embodiment ratings as a function of time and condition. First, we tested if the conditions gave rise to equal levels of embodiment during the embodiment phase by a Bayesian ANOVA. The results indicated an overall increase of the ratings over time, BF_01_ < 0.01 (BF_10_ > 100.00), suggesting that embodiment had occurred across all conditions. Importantly there was substantial evidence against an impact of condition, BF_01_ = 14.22, and against an interaction, BF_01_ = 95.23.

In our main analysis, we focused on the ratings 30 s before the intervention (pre), right after the intervention (post), and at the end of the disembodiment phase (end). First, we analyzed the immediate impact of the intervention by comparing pre- and post-ratings over all three conditions in a two-way ANOVA. There were significant main effects of time (pre vs. post), *F*(1,31) = 13.43, *p* < .001, η_p_^2^ = 0.30, and condition (active vs. no movement vs. disruption), *F*(2,62) = 17.50, *p* < .001, η_p_^2^ = 0.36. Crucially, the interaction was significant as well, *F*(2,62) = 34.43, *p* < .001, η_p_^2^ = 0.53, ε = 0.80 (with ε indicating the correction factor according to Greenhouse and Geisser’s method if sphericity could not be assumed). Second to assess the final outcome of each intervention, we compared post- and end-ratings over all conditions. We observed a significant main effect of time (post vs. end), *F*(1,31) = 8.13, *p* = .008, η_p_^2^ = 0.21, and condition, *F*(2,62) = 39.53, *p* < .001, η_p_^2^ = 0.56, which were qualified by a robust interaction, *F*(2,62) = 7.93, *p* < .001, η_p_^2^ = 0.20.

### Contrasts and follow-up analyses

We had postulated a marked decrease in ratings from right before the intervention (pre) to right after (post) only for the disruption condition. Indeed, a *t*-test comparing pre- and post-ratings for the disruption condition was significant, *t*(31) = 5.51, *p* < .001, *d*_z_ $=\left|t\right|/\sqrt{n}$ = 0.97, and a follow-up analysis of the participants’ individual scores suggested that this decrease occurred with a similar magnitude for most participants (see Fig. S2 in the Supplementary Material). Neither hypothesis had predicted a pronounced change for the active condition, and a corresponding Bayesian *t*-test pointed to equality of the corresponding pre- and post-ratings, BF_01_ = 4.49. The no-movement condition showed a numerical decrease already for the first rating, which however, was not significant, *t*(31) = 1.55, *p* = .131, *d*_z_ *=* 0.27, BF_01_ = 1.79.

Follow-up Bayesian tests further confirmed our expectation of similar post- and end-ratings in the active condition, BF_01_ = 3.47, as well as in the disruption condition, BF_01_ = 3.04. Crucially, this analysis also indicated a sizeable reduction of embodiment from post to end in the no-movement condition, *t*(31) = 4.41, *p* < .001, *d*_z_ *=* 0.78. We followed up on this observation by testing the fit of different regression models (linear, cubic, exponential, and logarithmic) to the data of the no-movement condition using mixed-effects modelling (via the lme4 package version 1.1-23 for R). These analyses implemented time as a fixed effect and participant as a random effect, and we computed explained variance via the MuMIn package version 1.43.17 in R. The data were best explained in terms of the logarithmic model, conditional R^2^ = .63 (marginal R^2^ = 0.05), and the linear model, conditional R^2^ = .62 (marginal R^2^ = 0.05; see Fig. 1C for a distribution of the slope coefficients of this model).

## Discussion

The current data show that newly embodied body parts gradually fade from the body representation unless they are maintained continuously by multisensory matching. This favors the updating model over the persistence model. Additionally, we show that a single instance of (potentially threatening) visuo-tactile mismatch - the impact of a hammer on the rubber hand but not on the real hand - is sufficient to decrease embodiment instantaneously.

Perhaps surprisingly, the results further suggest that the rubber hand did not become disembodied completely in either condition: Average ratings levelled out at the middle of the rating scale towards the end of the disembodiment phase, i.e., at a score of 5 out of 10 while the rating scale had been anchored at 3 to indicate that participants could imagine that the hand belonged to them. The disembodiment effects observed here are thus relative, not absolute, and additional factors likely promote residual feelings of ownership.^2^ One of these factors is the similarity of the rubber hand’s appearance and its orientation on the table relative to the participant’s real hand. Indeed, merely perceiving a rubber hand with an anatomically plausible orientation can be sufficient to instill embodiment (Fusco et al., 2020). The observation of gradually increasing embodiment ratings in the embodiment phase together with the gradual decrease observed the disembodiment phase of the no-movement condition suggests that mere visual information is not sufficient to yield strong embodiment in the chosen setup, however. Additional integration of converging multisensory information is thus necessary to integrate a new entity in the body representation.

The temporal evolution of embodiment ratings in the individual conditions further suggests that the maintenance of ownership is sensitive to different manipulations of multisensory input. In the no-movement condition ownership gradually declined over time. Thus, maintaining ownership over a newly embodied limb requires congruent and continuous multisensory input. This is in line with previous accounts of the rubber-hand illusion, which emphasize the role of bottom-up information in the generation of ownership (Makin, Holmes, & Ehrsson, 2008). In contrast to this, the disruption condition revealed that a sudden non-congruent input (a mismatch between visual and tactile information) reduces the sense of ownership instantaneously. This difference in the temporal pattern of ownership when facing different manipulations (absence of confirmatory signals vs. presence of incongruent signals) may speak for different mechanisms underlying these processes. It has been hypothesized that the generation of ownership by multisensory integration versus its loss (or disownership) is mediated by different cognitive and neural processes (De Vignemont, 2011), with several neuroimaging studies on healthy participants experiencing ownership illusions and on brain-lesioned patients suffering from asomatognosia (i.e., loss of ownership) indicating different cortical regions (premotor-parietal vs. insular regions; Brozzoli, Gentile, & Ehrsson, 2012; Karnath & Baier, 2010). These findings contrast with observations that similar premotor-parietal circuits are involved in embodying a new entity and in removing this entity following multisensory conflict (Gentile, Guterstam, Brozzoli, & Ehrsson, 2013). Further studies are thus needed to investigate not only the perceptual and neural mechanisms leading to the formation of ownership, but also the disruption of these processes leading to disownership.

In a similar vein, focusing on disembodiment for newly embodied entities might shed new light on empirical markers that have commonly been taken as implicit measures of embodiment. For instance, participants show robust changes in skin conductance responses when the rubber hand suddenly gets “injured” by contact with a sharp object (Armel & Ramachandran, 2003; Yuan & Steed, 2010). Even though these physiological changes might indeed index embodiment, they might at the same time reflect immediate disembodiment of the entity in question, thus yielding a rapid update of the body representation to its pre-experimental state.^3^

The present updating model further suggests that subtle signs of disembodiment might be observed for real body parts when there is no multisensory updating. Evidence for such disembodiment has been reported in the literature, for example, in terms of a reduced temperature of the hidden real hand during the rubber-hand illusion (Folegatti et al., 2009; Moseley et al., 2008; Tieri et al., 2017). Questionnaire data and phenomenological assessments also support the idea that a newly embodied limb may at times fully replace its original counterpart (Kannape et al., 2019; Kilteni, Normand, Sanchez-Vives, & Slater, 2012; Lane et al., 2017; Lewis & Lloyd, 2010; Longo et al., 2009; Valenzuela Moguillansky et al., 2013). In situations in which there is no salient alternative, however, body representations have been shown to be highly persistent as suggested by experiences of phantom limbs in patients after amputation (Flor et al., 1995; Ramachandran et al., 1995). It thus seems as if the updating model mainly applies to situations in which a new entity has just been incorporated in the body representation. It is an interesting and relevant question how much experience it takes to integrate an alien body part firmly into the body representation so that it stands the test of time more steadily than the newly integrated body parts as studied in the present experiment.

Finally, the present focus on newly embodied entities also comes with implications for designing human-machine interfaces in virtual and augmented reality scenarios. Embodiment is closely related to immersion and presence in virtual environments (Jung & Hughes, 2016; Tanaka, Nakanishi, & Ishiguro, 2015; Waltemate, Gall, Roth, Botsch, & Latoschik, 2018), and these phenomena are usually what educational programs or game companies aim to achieve in their outlets. Our data indicate that in order for embodiment not to fade, users must be provided with an opportunity to continuously revalidate their body representation, for instance by being able to move the virtual hand or by other means of detecting matching visual and tactile information. Additionally, our results indicate that even a single instance of meaningful visuo-tactile discrepancy has an immediate and profound impact on subjective embodiment. It still stands to question, however, how easily a limb that has been rejected in that manner is reintegrated into the body representation if synchronous feedback is reinstated. Exploring such re-integration will not only advance current theories on body representations but will also provide applied potential for therapeutic approaches to loss of ownership in clinical conditions such as asomatognosia and somatoparaphrenia (Giummarra, Gibson, Georgiou-Karistianis, & Bradshaw, 2008).

## Supplementary Information

ESM 1 (DOCX 422 kb)

## Footnotes

1 In addition to the main discrepancy of visual and tactile feedback introduced by seeing but not feeling how the hammer hits the embodied rubber hand, participants might also flinch in response to the hammer intervention. We did not discourage nor monitor such flinching movements as they would only increase the discrepancy between multisensory signals, which is precisely what the hammer intervention aimed at.

2 An additional, not mutually exclusive option, is that ratings in the later stages of a trial might be influenced by anchoring effects in that having rated a high number by the end of the embodiment phase increases the likelihood of giving relatively high ratings during the disembodiment phase (Tversky & Kahneman; 1974; Wilson et al., 1996). Potential anchoring effects would apply to all conditions in a similar way, though, because the conditions came with closely matched ratings during the embodiment phase (i.e., up to the point of the intervention).

3 We thank an anonymous reviewer for suggesting this point.
