# Supplementary material for: How to lose a hand: Sensory updating drives disembodiment
Source: Psychon Bull Rev. 2020 Dec 9;28(3):827–33. doi: 10.3758/s13423-020-01854-0 (PMC8219564; doi:10.3758/s13423-020-01854-0)
Supplement: Supplementary file 1 — (DOCX 422 kb) [file 13423_2020_1854_MOESM1_ESM.docx]

**Supplementary Material**

**Pre-experiment**

Setup and procedure were similar to the main experiment, with the exception that participants only performed one triplet of conditions while rating their subjective embodiment. In another triplet of conditions they judged the vertical position of their real hand every 30 seconds instead to capture proprioceptive drift. We focus on the subjective rating data here because these results were the basis for the present study (complete raw data are available at https://osf.io/ew6k9/). All analyses were as in the main experiment.

We collected a sample of 42 participants, of which we excluded 15 non-responders, who reported an embodiment score of 3/10 or less at a time when embodiment should be firmly established (pre-rating) once during the experiment – not twice, as in the main experiment, given they only performed only one triplet of conditions for the embodiment ratings. The remaining sample was predominantly female (22 female, 5 male), all but 3 right handed, and had a mean age of 20.96 years (18-28, SD: 2.61). Figure S1 summarizes the main results; inferential statistics are compiled in Tables S1 and S2.

**Table S1.** Test statistics for all computed analyses of variance (ANOVAs)

|  | *F* | *df*_test_ | *df*_error_ | *p* | η_p_^2^ | ε | BF_01_ |
| --- | --- | --- | --- | --- | --- | --- | --- |
| Bayesian ANOVA over the ratings of the embodiment phase in all three conditions | | | | | | | |
| time |  |  |  |  |  |  | 0.22 |
| condition |  |  |  |  |  |  | 12.35 |
| time * condition |  |  |  |  |  |  | 51.78 |
| Two-way ANOVA with the factors time [pre vs. post] and condition | | | | | | | |
| time | 11.29 | 1 | 26 | .002 | 0.30 |  |  |
| condition | 4.45 | 2 | 52 | .016 | 0.15 |  |  |
| time * condition | 12.29 | 2 | 52 | < .001 | 0.32 | 0.73 |  |
| Two-way ANOVA with the factors time [post vs. end] and condition | | | | | | | |
| time | 0.10 | 1 | 26 | .758 |  |  |  |
| condition | 13.55 | 2 | 52 | < .001 | 0.34 |  |  |
| time * condition | 5.80 | 2 | 52 | .005 | 0.18 |  |  |

*Note.* For the Bayesian ANOVA we report the Bayes Factor in favor of the null hypothesis of equality of conditions (BF_01_). Estimates for ε is only reported when sphericity could not be assumed (based on Mauchly’s test), and test statistics are corrected accordingly by the method of Greenhouse and Geisser.

**Table S2.** Test statistics for all computed *t*-tests

|  | | *t* | *df* | *p* | *d* | BF_01_ |
| --- | --- | --- | --- | --- | --- | --- |
| active | pre vs. post |  |  |  |  | 4.32 |
|  | post vs. end |  |  |  |  | 1.78 |
| no-movement | pre vs. post |  |  |  |  | 3.76 |
|  | post vs. end | 2.57 | 26 | .016 | 0.49 |  |
| disruption | pre vs. post | 3.98 | 26 | < .001 | 0.77 |  |
|  | post vs. end |  |  |  |  | 2.82 |

*Note.* For the Bayesian *t*-tests, only the Bayes Factor in favor of the null hypothesis of equality of conditions (BF_01_) is reported.


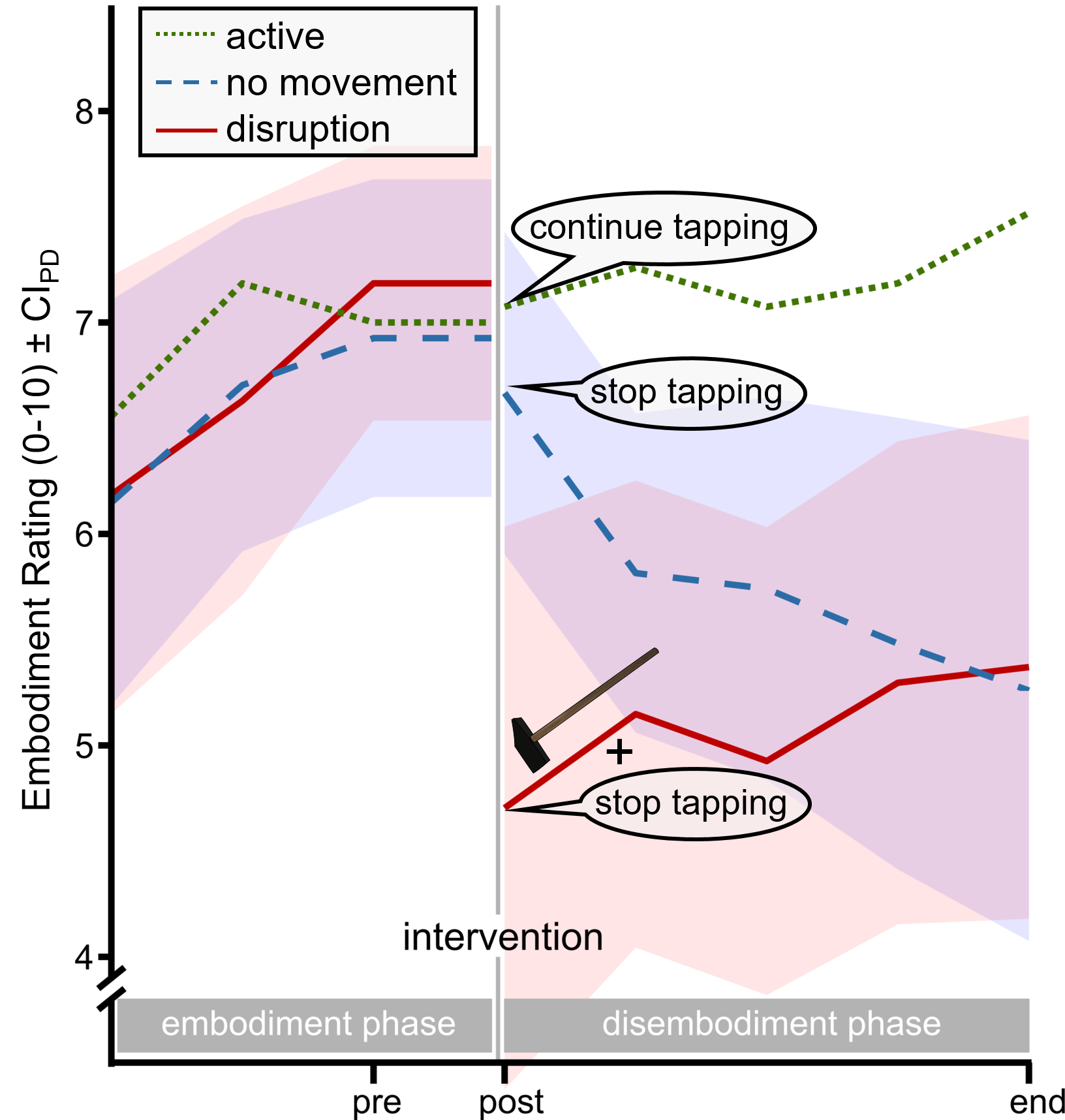


**Figure S1.** Results of the pre-experiment. Shaded areas indicate 95% confidence intervals of paired differences (CI_PD_) relative to the active condition.

**Embodiment scale**

The original wording of the anchors of the embodiment question was as follows:

0 – “Ich spüre keinen Bezug zwischen mir und der Hand.“ (“*I feel no relation between myself and the hand”)*

3 – „Ich könnte mir vorstellen, dass die Hand zu mir gehört.“ (*“I could imagine, that the hand belongs to me.”)*

7 – „Ich habe das Gefühl, dass die Hand Teil meines Körpers ist.“ (“*I have the feeling, that the hand is part of my body.”)*

10 – „Ich habe das Gefühl, dass die Hand meine eigene Hand ist.“ (“*I have the feeling that the hand is my own hand*.”)

**By-participant plots**

Figure S2 shows individual data points for the comparison of pre- and post-ratings in the disruption condition. The immediate but imperfect drop in embodiment ratings of about two points on the ratings scale occurred rather consistently throughout the sample (with only few participants showing no or a slightly reversed pattern) and it also occurred irrespective of the participants’ initial level before the intervention.


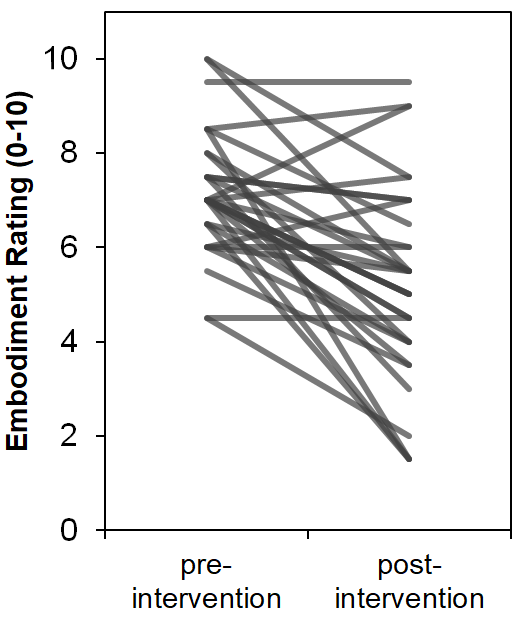


**Fig. S2.** Individual data points for the comparison of pre- and post-ratings in the disruption condition.
